# Supplementary material for: Splice-Junction-Based Mapping of Alternative Isoforms in the Human Proteome
Source: Cell Rep. Author manuscript; Available in PMC 2020 Jan 15. (PMC6961840; doi:10.1016/j.celrep.2019.11.026)
Supplement: 2 [file NIHMS1546469-supplement-2.zip › DF1/PXD010154/Lung/Lung_49_KRT18_SADSTNYRSLGSVQAPSYGARPVSSAASVYAGAGGSG.pdf]

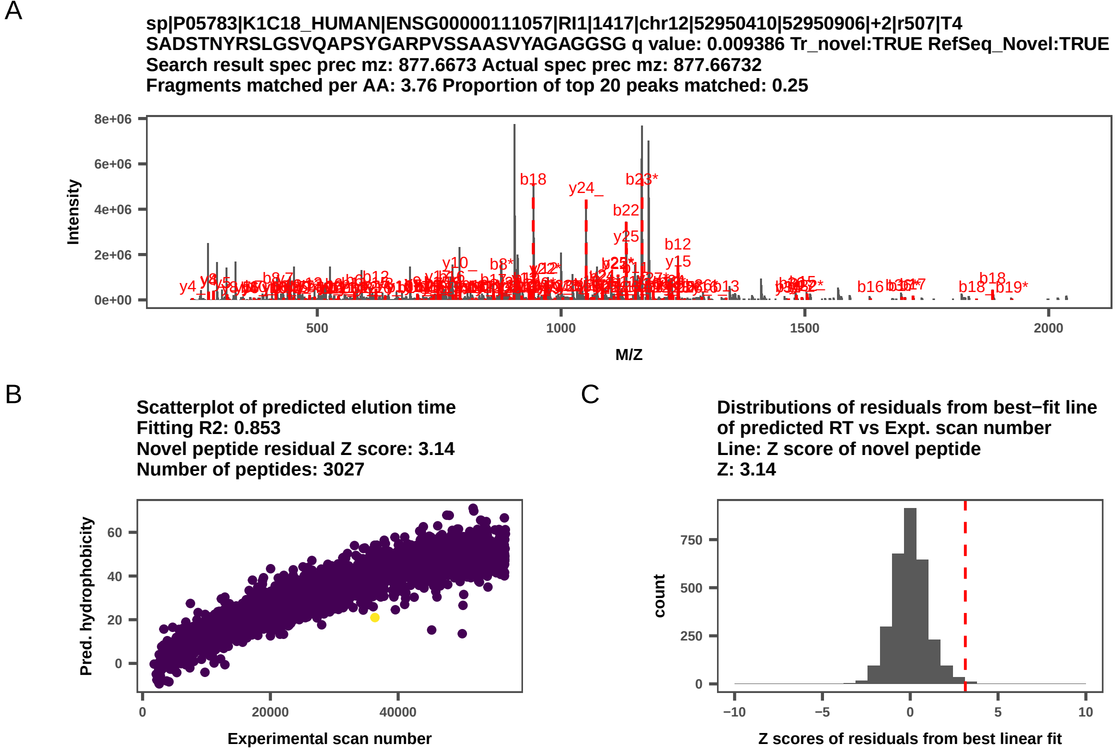
A
sp|P05783|K1C18_HUMAN|ENSG00000111057|RI1|1417|chr12|52950410|52950906|+2|r507|T4
SADSTNYRSLGSVQAPSYGARPVSSAASVYAGAGGSG q value: 0.009386 Tr_novel:TRUE RefSeq_Novel:TRUE
Search result spec prec mz: 877.6673 Actual spec prec mz: 877.66732
Fragments matched per AA: 3.76 Proportion of top 20 peaks matched: 0.25
Intensity
8e+06
6e+06
4e+06
2e+06
0e+00
b18
y24_
b23*
b22
y25
b12
y15
y10_
b8*
y12*
b13
b16
b18
b19*
500
1000
1500
2000
M/Z
B
Scatterplot of predicted elution time
Fitting R2: 0.853
Novel peptide residual Z score: 3.14
Number of peptides: 3027
Pred. hydrophobicity
60
40
20
0
0
20000
40000
Experimental scan number
C
Distributions of residuals from best−fit line
of predicted RT vs Expt. scan number
Line: Z score of novel peptide
Z: 3.14
count
750
500
250
0
−10
−5
0
5
10
Z scores of residuals from best linear fit
